# Bat trait, genetic and pathogen data from large-scale investigations of African fruit bats, *Eidolon helvum*

**DOI:** 10.1038/sdata.2016.49

**Published:** 2016-08-01

**Authors:** Alison J. Peel, Kate S. Baker, David T. S. Hayman, Richard Suu-Ire, Andrew C. Breed, Guy-Crispin Gembu, Tiziana Lembo, Andrés Fernández-Loras, David R. Sargan, Anthony R. Fooks, Andrew A. Cunningham, James L. N. Wood

**Affiliations:** 1 Department of Veterinary Medicine, University of Cambridge, Cambridge CB3 0ES, UK; 2 Institute of Zoology, Zoological Society of London, Regent’s Park, London NW1 4RY, UK; 3 Environmental Futures Research Institute, Griffith University, Brisbane, Queensland 4111 Australia; 4 Wellcome Trust Sanger Institute, Hinxton CB10 1SA, UK; 5 Institute for Integrative Biology, University of Liverpool, Liverpool L69 7ZB, UK; 6 Molecular Epidemiology and Public Health Laboratory, Hopkirk Research Institute, Massey University, Private Bag, 11 222, Palmerston North 4442, New Zealand; 7 Wildlife Division, Ghana Forestry Commission, Accra, Ghana; 8 University of Ghana, Faculty of Animal Biology and Conservation Science, Box LG 571, Legon, Accra, Ghana; 9 Animal and Plant Health Agency (APHA), Addlestone, Surrey KT15 3NB, UK; 10 Faculté des Sciences, Université de Kisangani, 4, Avenue Kithima, commune Makiso, BP 2012, Kisangani, République Démocratique du Congo; 11 Institute of Biodiversity, Animal Health and Comparative Medicine, College of Medical, Veterinary and Life Sciences, University of Glasgow, Graham Kerr Building, Glasgow, G12 8QQ, Scotland; 12 Museo Nacional de Ciencias Naturales, CSIC, José Gutiérrez Abascal 2, Madrid 28006, Spain

**Keywords:** Population dynamics, Ecological epidemiology, Genetic variation

## Abstract

Bats, including African straw-coloured fruit bats (*Eidolon helvum*), have been highlighted as reservoirs of many recently emerged zoonotic viruses. This common, widespread and ecologically important species was the focus of longitudinal and continent-wide studies of the epidemiological and ecology of Lagos bat virus, henipaviruses and Achimota viruses. Here we present a spatial, morphological, demographic, genetic and serological dataset encompassing 2827 bats from nine countries over an 8-year period. Genetic data comprises cytochrome *b* mitochondrial sequences (*n*=608) and microsatellite genotypes from 18 loci (*n*=544). Tooth-cementum analyses (*n*=316) allowed derivation of rare age-specific serologic data for a lyssavirus, a henipavirus and two rubulaviruses. This dataset contributes a substantial volume of data on the ecology of *E. helvum* and its viruses and will be valuable for a wide range of studies, including viral transmission dynamic modelling in age-structured populations, investigation of seasonal reproductive asynchrony in wide-ranging species, ecological niche modelling, inference of island colonisation history, exploration of relationships between island and body size, and various spatial analyses of demographic, morphometric or serological data.

## Background & Summary

**Figure 1:**
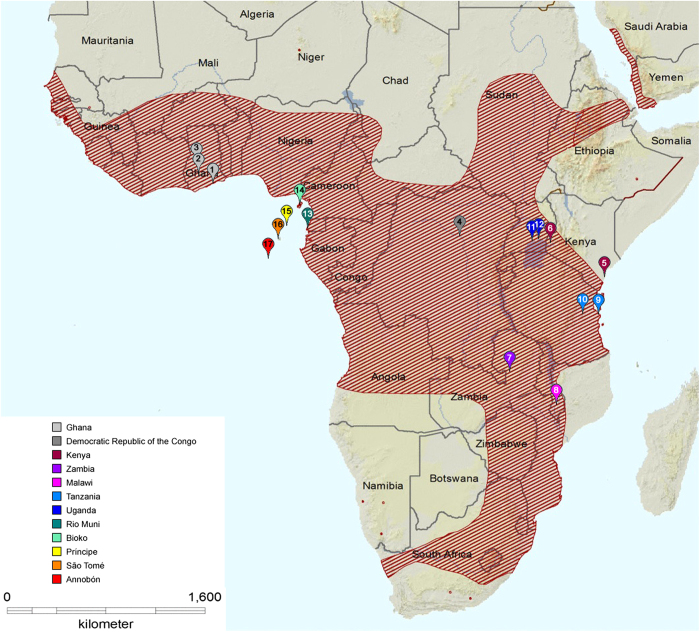
Map showing location of *E. helvum* sampling locations for genetic and serological analyses. Shading represents the distribution range of E. helvum. Sampling locations are numbered as in Table 1 (available online only). Adapted with permission from Mickleburgh *et al.*^43^ and Peel *et al.*^12^.

The straw-coloured fruit bat (*Eidolon helvum*) is a common, widely distributed, migratory species, occurring across sub-Saharan Africa and some offshore islands (Fig. 1)^1,2^. Since 2007, investigations into the epidemiology and ecology of zoonotic viral infections in *E. helvum* have been undertaken via longitudinal sampling of wild populations in Ghana. Complementing this, between 2008–2011 and in 2014, cross-sectional sampling events were undertaken to determine the genetic population structure of *E. helvum*, and to assess whether the serological findings in Ghana were representative across the species’ range (Fig. 1).

Four viruses were the focus of our serological surveys in *E. helvum* bats: Lagos bat virus (LBV), African henipaviruses, Achimota virus 1 (AchPV1) and Achimota virus 2 (AchPV2). Lagos bat virus is one of at least 15 known species in the *Lyssavirus* genus^3^ and has been isolated from *E. helvum* on multiple occasions^4,5^. An African henipavirus is still yet to be isolated, however a full genome sequence has been obtained (putative name: African bat henipavirus Eid_hel/GH-M74a/GHA/2009 (M74))^6^. Achimota viruses 1 and 2 are closely related rubulaviruses for which serological evidence suggestive of spillover to humans in Africa exists^7^.

The specific aims of the data collection were to:

- Investigate whether antibodies to LBV, henipaviruses and Achimota viruses are present in *E. helvum* across its continental and island range, and to explore the antibody dynamics where possible.
- Describe the genetic metapopulation structure of *E. helvum* using a combination of mitochondrial (mtDNA) and microsatellite markers.
- Gather information on *E. helvum* distribution and seasonal patterns of reproduction.
- Combine results from these multidisciplinary studies to make inferences about virus transmission dynamics, and ultimately make inferences on the spillover risk to human populations.

**Figure 2:**
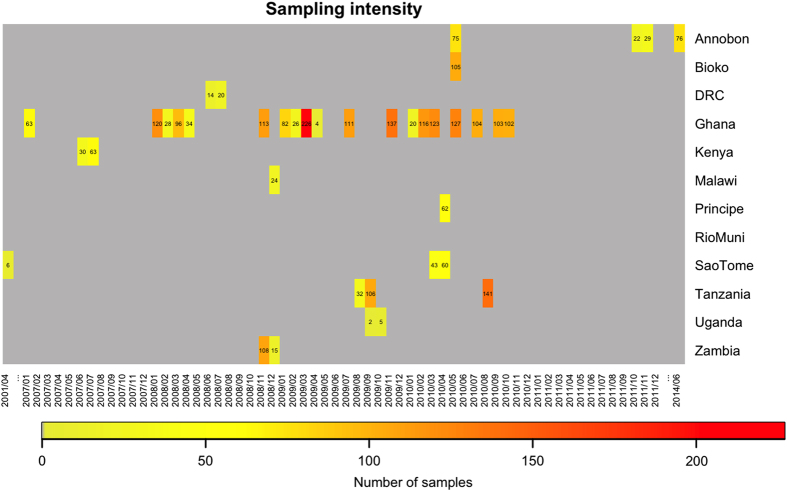
Sampling intensity per month, by country. Red represents high sampling intensity and the numbers of samples collected per month is recorded within each grid cell. Records in the database with unknown collection date are not represented here (nine from Annobón, seven from Bioko, 14 from Príncipe, 10 from Rio Muni and 13 from São Tomé).

Samples in this dataset are from 2827 bats from nine countries over an 8-year period (Fig. 2, Table 1 (available online only)). Raw data comprises spatial (roost location), seasonal (timing of sampling and seasonal birth pulses), morphological (forearm length, body weight), demographic (age, sex, reproductive status, mother-offspring relationships) and identification (individually numbered thumb-band) components. Data generated includes genetic characterisation (mtDNA sequencing and microsatellite genotyping) and serological assay results (for LBV, henipaviruses and Achimota viruses).

Multiple publications have arisen from these data, however many aspects remain unexplored. Demographic analyses have estimated birth and survival rates^8,9^, and explored the effect of hunting on the latter^9^. Variations in roost composition have suggested a fission-fusion social structure^9^. Serological analyses have identified: the presence of antibodies against LBV, henipaviruses and Achimota viruses in *E. helvum* in Ghana^7,10,11^ and more broadly across the species’ range, including isolated off-shore islands^7,12,13^; that these viruses circulate endemically in *E. helvum* in Ghana, with evidence of horizontal transmission^7,14,15^; and that *E. helvum* bats previously infected with LBV can have long-survival post infection^16^. The henipavirus dataset was used to develop a Bayesian method to determine appropriate cutoffs for serological assays^17^. Population genetic analyses identified that *E. helvum* are panmictic across their continental range, but that genetically isolated populations exist on isolated islands^12^.

Other publications arising from these samples, but based on analyses not included here, include the development of a universal real-time assay and a pseudotype neutralisation assay for Lyssaviruses^18,19^, microsatellite loci characterisation^20^, estimation of divergence times between *Eidolon* sister species^21^, inference of movement ecology based on stable isotope ratios^22^, demonstration of Ebola antibodies^16,23^, identification of multiple novel viruses^24^, and novel Bartonella species in bat flies collected from *E. helvum*^25,26^.

This dataset contributes a substantial volume of data on the ecology of *E. helvum* and its viruses and will be valuable for a wide range of studies. In particular, an age-specific dataset such as this is rare and valuable for wildlife, especially bats. Further analyses could include viral transmission dynamic modelling in age-structured populations, including the use of cutting-edge Bayesian approaches to address complex epidemiological questions^27^; time-series analyses on 5 years of wild henipavirus serological data from the same study site in Ghana (*n*=1486 data points), investigation of seasonal reproductive asynchrony in wide-ranging species; ecological niche modelling; inference of island colonisation history, exploration of relationships between island and body size; and various spatial analyses of demographic, morphometric or serological data. Field samples (e.g. serum, blood cells, urine, skin samples) and extracted DNA from individual bats in this dataset exist in storage and the authors are open to collaborative requests to undertake further analyses.

## Methods

These methods are expanded and modified versions of descriptions in our previous publications, as cited in each section below. All associated data can be found in ‘Eidolon helvum data 2007–2014.csv’ [Data Citation 1].

### Capture and Data Collection

Capture and sampling information has been described previously e.g. (refs 10,12). Sampling locations comprised 13 *E. helvum* roosting sites in continental Africa, and 14 in the four main islands in the Gulf of Guinea (Fig. 1, further detail in Table 1 (available online only)). In the majority of locations, data are from a single sampling event (sometimes comprising multiple sampling sessions within a one month period). Repeated sampling was conducted in Ghana (multiple sampling events per year over four years), Tanzania (one sampling event per year over two years) and Annobón (three sampling events over 4 years) as these locations were the focus of specific research studies. All fieldwork was undertaken under permits granted by national and local authorities (listed in Acknowledgements) and under ethics approval from the Zoological Society of London Ethics Committee (WLE/0489 and WLE/0467), using field protocols which followed ASM guidelines^28^. Bats were captured at the roost with mist nets (6–18 m; 38 mm) as they departed the roost site at dusk, or returned at dawn. Except for a proportion of bats that were euthanased for virological studies (*n*=238), bats were released following sampling. Additional samples and data were obtained from other research groups (*n*=152) and in collaboration with local hunters in São Tomé (*n*=102), where bats are hunted for human consumption.

Personal protective equipment (long clothing, face masks, eye protection and gloves) was worn during sample collection. Morphometric and demographic details were recorded from bats under manual restraint. Female reproductive status was assigned as non-reproductive, pregnant, or lactating, according to the descriptions provided in Table 2. The phase in the reproductive cycle (i.e. the time in months between the sampling date and the beginning of the last birthing season) was estimated based on published data and the pregnancy status of females (foetal size, assuming a true gestation period of 4 months (Mutere 1965)) or degree of juvenile development during sampling.

**Figure 3:**
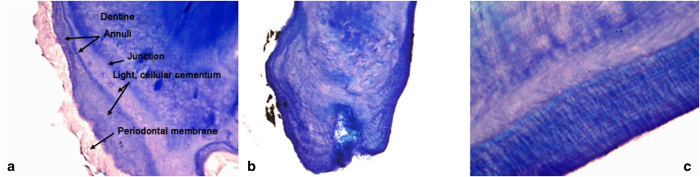
Histological sections of upper canine teeth from *E. helvum* for cementum age analysis (Giemsa stain). Photographs and captions courtesy of Gary Matson, Matson’s Laboratory, MT, USA. Each age estimation was scored with a certainty code: A: highest certainty of reported age, B: histological evidence supported a given age result±0.5–1.5 years, or C: tooth or section quality was too compromised to accurately age. (**a**) Bat ID 424. Cementum age 2, certainty code A. 100X. The tooth was in excellent histological condition, as indicated by the presence of periodontal membrane and good differential staining between annuli and light cementum. (**b**) Bat ID 62. Cementum age 6, certainty code A. 100X. Annuli are complex, with at least two components each year. A key feature of age analysis is resolving uncertainty about whether complex annuli or individual components are being used as age indicators. (**c**) Bat ID 44. Cementum age 13, certainty code B (13–15 yrs). 400X. The root tip of this tooth had been broken off during extraction. Missing cementum complicates age analysis, reducing the evidence available for evaluating whether annuli observed at one point may be clearly identifiable as components of complex annuli at another point.

Age was assessed by morphological characteristics (Table 3) and all individuals were placed into one of four age classes: Neonate (N; <2mths), Juvenile (J; 2–<6 months), Sexually Immature (SI; 6–<24 months) or Adult (A; ≥24 months). For a subset of samples, the timing of sampling in relation to the birthing season permitted further classification of SI individuals into 6-month age groups SI.1, SI.2 and SI.3 (6 –<12, 12–<18, 18–<24 months, respectively). Additionally, for bats that were hunted or euthanased following capture, upper canine teeth were extracted, air dried and shipped to the USA (Matson’s laboratory, USA) for histological examination to assess the number of tooth cementum annuli present^29,30^. Following previous studies^31^, it was assumed that each observed cementum layer represented one year. Each age estimation was scored with a certainty code: A: highest certainty of reported age (51% of samples, e.g. Fig. 3a,b), B: histological evidence supported a given age result±0.5–1.5 years (46% of samples, e.g. Fig. 3c), or C: tooth or section quality was too compromised to accurately age (3% of samples).

Genetic and blood samples were collected under manual restraint. Wing membrane biopsies (4-mm) were placed into 70% alcohol. Up to 1 ml blood was collected from the propatagial vein using a citrated 1 ml syringe and placed into a plain 1.5 ml eppendorf tube.

### Molecular methods

Molecular methods have been described previously^12,20^. Genomic DNA was extracted from *E. helvum* tissues (predominantly wing membrane biopsies, but also liver and muscle samples, all stored in ethanol) using DNeasy Blood and Tissue Kits (QIAGEN Ltd., Crawley, West Sussex, UK). DNA was quantified using Quant-iT PicoGreen dsDNA kits (Molecular probes, UK), and later using a Nanodrop ND-1000 Spectrophotometer (Thermo Fisher Scientific, UK) and diluted to a standard concentration.

Twenty *E. helvum* loci developed in a previous study^20^ were quality-checked using a subset of samples. Loci E and Ae were discarded due to difficulty in scoring or high error rates and data from locus Ag were re-binned and re-scored, correcting earlier issues with allelic dropout. In total, 170 continental and 385 island samples were run as multiplex PCRs at 18 loci (TSY, FWB, MNQX, AgPK, AcAfAi, AdAh) in 10 μl PCRs, containing 4ng template DNA, 0.2 μM of each primer, and 5 μl Type-it Multiplex PCR Master Mix (QIAGEN Ltd.). Positive and negative controls were included on each plate and amplification was performed using the following conditions: 5 min at 94 °C; 30 cycles of 30 s at 95 °C, 90 s at 57 °C, and 30 s at 72 °C; then 30 s at 60 °C. Genotyping was performed by capillary electrophoresis using a Beckman CEQ 8000 (Beckman, UK). Allele sizes were scored automatically prior to manual verification. Genotyping data from 18 loci are provided in ‘Eidolon helvum data 2007–2014.csv’ [Data Citation 1]. Loci B has previously been identified as being X-linked^20^.

Fragments of the mitochondrial DNA cytochrome b (cytb) gene were amplified from continental samples by PCR using the generic primers L14722 (5′- CGA AGC TTG ATA TGA AAA ACC ATC GTT G)^32^ and H15149 (5′- AAA CTG CAG CCC CTC AGA ATG ATA TTT GTC CTC A)^33^ in 20 μl reactions, containing 0.1–1 ng template DNA, 0.2 μM of each primer, 0.25 mM of each dNTP, 1.5 mM MgCl_2_, 0.25 μl of *Taq* polymerase (Invitrogen), and 0.2 μl 10X reaction buffer and with the following conditions: 5 min at 94 °C; 40 cycles of 1 min at 93 °C, 1 min at 54 °C, and 2 min at 72 °C; then 7 min at 72 °C. Although these generic primers were adequate with continental samples (8% PCR failure), amplification from isolated Gulf of Guinea island samples was less successful (48% PCR failure). Shortened primers (EhM2814 (5′- GCT TGA TAT GAA AAA CCA TCG TTG) and EhM2815 (5′- CAG CCC CTC AGA ATG ATA TTT GT) resulted in successful amplification when using Microzone MegaMix-Gold reagent (Microzone Ltd, UK). PCRs were performed in 20 μl reactions, containing 2 ng template DNA, 0.25 μM of each primer, and 10 μl MegaMix-Gold, using the following conditions: 5 min at 95 °C; 33 cycles of 30 sec at 95 °C, 30 sec at 53 °C, and 45 sec at 72 °C. PCR products were checked by gel electrophoresis on 1% agarose gels, purified using Exosap-IT clean-up (USB Europe, Germany) and sequenced in both directions on an ABI 3730xl DNA Analyser, (Applied Biosystems). Paired sequences were edited and aligned using the STADEN Package v1.6 (ref. 34). Multiple sequence alignment was performed using default settings in T-COFFEE^35^. Sequences were checked manually and trimmed to a standard length (397 bp) in JALVIEW v2 (ref. 36). No sequence differences were detected in 38 samples sequenced using both primer pairs, so data were combined.

Data from 608 and 544 individuals is available for cytb and microsatellite analyses (at 18 loci), respectively (Table 1 (available online only)).

### Serological analyses

Serological methods have been described previously^7,10–14^.

A modified fluorescent antibody virus neutralization (mFAVN) assay using the LBVNig56 isolate was used to detect neutralising antibodies against LBV^10,37^. Samples were tested in duplicate using threefold serial dilutions (representing reciprocal titres of 9, 27, 81, and 243–19,683). Human rabies immunoglobulin, LBV-positive rabbit serum, and rabies-vaccinated mouse serum were used as positive controls and negative rabbit and mouse serum were negative controls. Titres were considered positive at IC100 endpoint reciprocal dilutions >1: 9 (100% neutralisation of virus).

**Figure 4:**
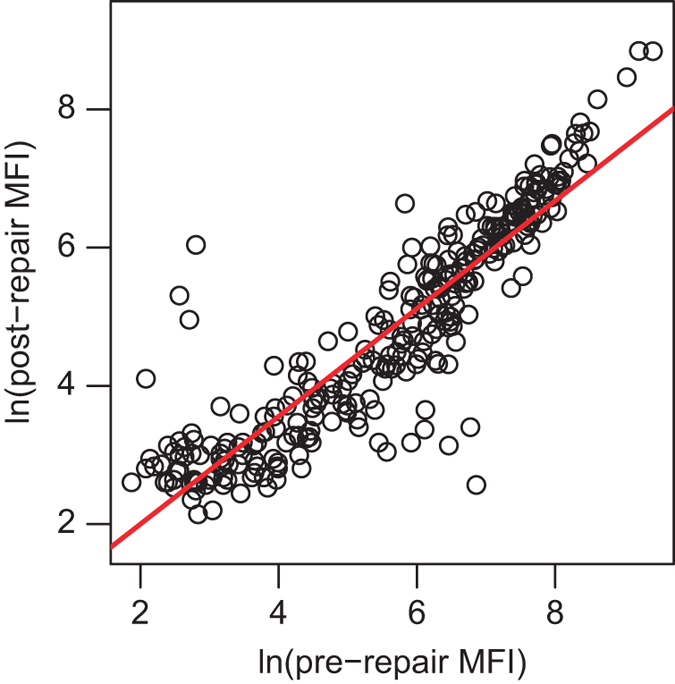
Correlation between *ln*(MFI) values pre- and post-repair of the Luminex machine used to run the assays, based on 293 samples. The linear regression line is in red. (*R*^2^0.81, F-statistic: 1306 (1, 296), *P*<2.2e–16).

Henipavirus antibodies detected in African fruit bat samples using virus neutralisation assays, multiplexed microsphere assays and pseudotype assays developed to target other known henipaviruses (Hendra and Nipah viruses) and are presumed to represent cross-neutralisation or cross-reactivity^12^. Here, Luminex multiplexed microsphere binding assays were used to detect antibodies against henipaviruses (HeV and NiV). In these assays, purified recombinant expressed henipavirus soluble G glycoproteins^38^ are conjugated to internally coloured and distinguishable microspheres, allowing multiplexing. For African bat samples, stronger results were consistently observed in NiV binding assays and virus neutralisation tests^13^, so only NiV binding assay results are included in the dataset. Binding results are outputted as median fluorescence intensity (MFI) values of at least 100 microspheres for each virus type. In mid-December 2010, major repair work was undertaken on the Luminex machine being used for serological analysis. A subset of samples that had been analysed before the repairs were repeated to calibrate results (*n*=293). MFI values pre- and post-repair work were significantly different, making the use of a single cutoff inappropriate^17^. Two approaches were taken to designate results as seropositive or seronegative. First, a Bayesian mixture model was applied as described in^17^. Cutoffs for pre- and post-repair work were determined so that samples above this cutoff were ≥ 99% likely to be in the seropositive distribution (MFI=156.1 and 127.5, respectively). Second, linear regression of pre- and post-maintenance *ln*(MFI) values demonstrated a significant linear relationship (Fig. 4, *R*^2^0.81, F-statistic: 1306 (1, 296), *P*<2.2e-16), and the variance decreases for higher MFI values (above the cutoff). Pre-maintenance MFI values were converted to post-maintenance values using the formula:

NEW_MFI=exp (0.7795774*ln(OLD_MFI)+0.4392832).

The Bayesian mixture model was applied to this transformed and combined data using the same method. From this analysis, MFI values >94.2 were ≥ 99% likely to be in the seropositive distribution. Results from the two methods were compared and the second method resulted in the highest congruence between pre- and post-maintenance paired results (congruence in 266/298 samples versus 250/298 samples for the first method), and these data were therefore used in the final dataset. Raw MFI values are available on request.

Antibodies against Achimota viruses 1 and 2 were detected using virus neutralisation assays^7^, with all testing in duplicate. Samples were diluted to 1:20 and incubated with 200 TCID50 of virus for 30 min at 37 °C prior to the addition of Vero cell suspension at an MOI equivalent to 0.01. Cell monolayers were assessed for evidence of virus neutralization 7 days post infection. Where sample volume permitted, positive samples were titrated in a 2-fold dilution series from 1: 20 to 1: 160 and retested using the same protocol.

## Data Records

The data are contained in a single comma-separated file (.csv format), entitled ‘Eidolon helvum data 2007–2014’ (Data Citation 1). Each row below the header represents an individual bat (*n*=2,827), and the columns (*n*=68) contain sample identifier information, demographic and morphometric data, and results of genetic and serological assays. Full descriptions of the column titles are included in the Table 4 (available online only).

## Technical Validation

### Molecular analyses

Recommendations for minimisation and assessment of errors that may occur during the sampling, DNA extraction, amplification, sequencing, genotyping and data analysis processes were followed where possible^39^ (Table 5).

As previously described^20^, microsatellite loci were tested for evidence of departure from Hardy–Weinberg equilibrium (HWE) and genotypic disequilibrium using FSTAT 2.9 (ref. 40), with appropriate Bonferroni corrections for multiple testing. All loci were analysed in MICRO-CHECKER^41^ to test for null alleles, stuttering and large allelic dropout as a cause of departure from HWE. Additionally, since Locus M displayed extremely low polymorphism (99.1% of individuals were homozygous for a particular allele), this locus was included in all PCR plates as a positive control and to determine inter-assay variability in allele fragment length. Error rates for microsatellite loci are reported in Peel *et al.*^20^ Inter-assay genotyping variability, measured by the variation in fragment length of the dominant allele of locus M on each plate, was low (range 134.32−134.66) across 27 runs and two control samples. Loci Y proved difficult to confidently bin due to alleles of single nucleotide difference and was therefore not included in the dataset.

Error rates for cytb analyses were assessed by replicate extractions (performed on 2.4% of samples), replicate PCR and sequencing reactions (performed on 8–14% of extracted samples), and by inclusion of positive and negative controls for all extractions and PCRs. Poor quality mtDNA sequence traces were excluded. Background PCR and sequencing error rates of the new *E. helvum* cytb primers EhM2814 and EhM2815 were assessed by running 70 replicates of a single sample. PCR and sequencing error rates were calculated at the base-pair level. Sequencing error rate was negligible (0–0.01%) across samples repeated in duplicate, and no substitutions were observed in the 70 replicate sequences obtained from a single sample (Table 6).

### Serological analyses

All serological assays included positive and negative controls. Samples were tested in duplicate (LBV and Achimota viruses) or with 100 replicates (henipaviruses). Further validation procedures for multiplexed microsphere binding assays are presented as part of the methods, above.

## Usage Notes

Users of these data are advised that importing the.csv data file (Data Citation 1) into Microsoft Excel can result in formatting errors, particularly with the column ‘Teeth.Age.Range’. Rather than opening the file with Excel (by double-clicking, for example), it is suggested that users instead select ‘File>Import>csv file >Delimited’, then select the ‘Teeth.Age.Range’ column and set the column data format as ‘Text’. Alternatively, importing and processing the data into the software ‘R’^42^ may be preferable.

## Additional Information

**How to cite**: Peel, A. J. *et al.* Bat trait, genetic and pathogen data from large-scale investigations of African fruit bats, *Eidolon helvum.* *Sci. Data* 3:160049 doi: 10.1038/sdata.2016.49 (2016).

## Supplementary Material



## Figures and Tables

**Table 1:** Overview of *Eidolon helvum* sampling locations and sample types used in this study

	**Ghana**	**Democratic Republic of the Congo**	**Kenya**	**Zambia**	**Malawi**	**Tanzania**	**Uganda**	**Rio Muni**	**Bioko**	**Príncipe**	**São Tomé**	**Annobón**	**TOTAL**
**Map ref**	1, 2, 3	4	5, 6	7	8	9, 10	11, 12	13	14	15	16	17	
**Sampling locations**	5.588 N, 0.182 W	0.528 N, 25.371 E	0.007 S, 34.605 E	12.589 S, 30.246 E	15.788 S, 35.011 E	6.801 S, 39.283 E	0.303 N, 32.554 E	*	3.747 N, 8.770 E	1.680 N, 7.390 E	0.239 N, 6.482 E	1.459 S, 5.645 E	
	6.700 N, 1.625 W		0.043 S, 34.410 E			6.800 S, 39.285 E	0.427 N, 33.201 E		3.752 N, 8.772 E	1.590 N, 7.337 E	0.341 N, 6.563 E	1.418 S, 5.621 E	
	7.647 N, 1.882 W		3.216 N, 40.119 E			6.823 S, 37.666 E	0.416 N, 33.204 E		*	*	0.286 N, 6.678 E	1.460 S, 5.630 E	
											0.048 N, 6.505 E	*	
											0.022 N, 6.517 E		
											0.043 N, 6.544 E		
											0.029 N, 6.532 E		
											0.252 N, 6.671 E		
											*		
**Sampling Years**	2007, 2008, 2009	2008	2007	2008	2008	2009, 2010	2009	1992	1992, 2010	1992, 2010	1992, 2010	1992, 2010, 2011, 2014	
**Collected by**	DTSH, KSB, AJP, ACB, AFL	GCG	IVK^5^	AJP	AJP	AJP	AJP	JJ^44^	AJP, AFL, JJ^44^	AJP, AFL, JJ^44^	AJP, AFL, JJ^44^	AJP, AFL, RCCDS, IRP, ACB, JJ^44^	
**Number of bats**	1735	34	93	123	24	279	7	10	112	76	122	211	**2826**
**Number of forearm measurements**	1454	34	92	42	22	234	1	0	105	60	101	202	**2347**
**Number of body weight measurements**	1540	34	92	30	16	248	1	0	105	25	103	202	**2396**
**Number of tooth ages**	87	0	0	0	0	93	0	0	24	34	76	2	**316**
**Number of Lagos bat virus results**	792	0	0	10	12	230	5	0	105	57	96	121	**1428**
**Number of henipavirus results**	1637	0	0	12	16	248	7	0	105	62	98	123	**2308**
**Number of Achimota virus 1 results**	157	0	0	0	0	25	0	0	0	21	31	10	**244**
**Number of Achimota virus 2 results**	157	0	0	0	0	25	0	0	0	21	31	10	**244**
**Number of cytochrome b sequences**	74	21	20	23	24	50	7	10	108	70	118	83	**608**
**Number of microsatellite genotypes**	20	21	20	20	24	49	7	9	111	75	115	83	**554**
Map references refer to numbers in Fig. 1.													
Persons involved in sample collection: DTSH (David T.S. Hayman), AJP (Alison J. Peel), KSB (Kate S. Baker), GCG (Guy Crispin Gembu), AFL (Andrés Fernández Loras), RCCDS (Ricardo Castro Cesar De Sa), IRP (Iñaki Rodriguez Prieto), ACB (Andrew C Breed), JJ (Javier Juste), IVK (Ivan Kuzmin). Table modified from Peel *et al.* Continent-wide panmixia of an African fruit bat facilitates transmission of potentially zoonotic viruses. Nature Communications, 2013 4, p3770													

*Exact sampling location for some samples not available.

**Table 2:** Reproductive status classifications for female *E. helvum*.

**Phase**	**Reproductive status**	**Abbreviation**	**Description**
1–2	Lactating	L	Neonate attached and suckling, or milk able to be expressed from mammary glands of females
3–8	Non-reproductive	NR	Not detectably pregnant on abdominal palpation
9	Very early pregnant	VEP	Uterine bulge palpable on abdominal palpation, up to 1cm diameter (detectable from~0.5 cm diameter with careful palpation)
10	Early pregnant	EP	Uterine bulge palpable on abdominal palpation, 1–2 cm diameter
11	Mid pregnant	MP	Uterine bulge and foetus palpable on abdominal palpation, but no obvious abdominal distension of the female (2-~3.5 cm)
12	Late pregnant	LP	Foetus palpable on abdominal palpation, with distension of the female's abdomen so that it is wider than the base of the ribs
Phase represents the estimated time in months since the beginning of the last birthing season.			

**Table 3:** Age classification system used for *E. helvum*

**Age class**	**Abbr.**	**Age (mths)**	**Timing of presence**	**Classification features**	
**Standard age classes**	**Extended age classes**				
Neonate	Neonate		<2	Up to 2 months after birth pulse	Suckling
Juvenile	Juvenile	J	2–<6	2–6 months after birth pulse	Small body size
Sexually Immature	SI	6 – <24		Adult size (or nearly adult size), but undeveloped nipples and genitalia compared with adults	
	Sexually Immature 1	SI.1*	6 – <12	6–12 months after birth pulse	Early in this period, distinguishable from SI.3 by smaller body size, and from Adults by lack of sexual development. In the last 3 months, females expected to be pregnant, but with undeveloped nipples.
	Sexually Immature 2	SI.2	12 –<18	Up to 6 months after birth pulse	Clearly distinguishable from juveniles by size and adults by lack of sexual development
	Sexually Immature 3†	SI.3*	18 – <24	6–12 months after birth pulse	Early in this period, distinguishable from SI.1 by larger body size, and from Adults by lack of sexual development. In the last 3 months this is less clear, however females expected to be pregnant with their first offspring (primiparous), but with undeveloped nipples.†
Adult	Adult	A	≥ 24	All year	Full body size within ranges expected for an adult. Females have developed nipples, males with developed testicles.

*SI.1 and SI.3 stages represent two different birth cohorts, overlapping in time. Where distinction between SI.1 and SI.3 was unclear during sampling and analysis of measurements, individuals were simply classified as ‘SI’ and omitted from extended age class analyses.

^†^SI individuals become sexually active during the last 4 months of their second year, however the highly synchronous and seasonal nature of the birthing period means that it is simpler for individuals to be recognised as sexually mature only after the beginning of the birthing pulse. While pregnant SI.3 (primiparous) individuals are therefore not technically sexually ‘immature’, by classifying them as such, they can be differentiated from adult females (>2 years of age).

**Table 4:** Descriptor codes for data file

**Column header name**	**Description**
Sample	Unique sample identifier, generally consisting of two-letter country identifier, and sample number
SamplingEventID	Sampling event identifier, consisting of two-letter country identifier, three-letter location identifier (see LocationID) and the date of sampling in the format yymmdd
Samplers	Initials of researchers who undertook sample collection. ACB (Andrew C Breed), AFL (Andrés Fernández-Loras), AJP (Alison J Peel), DTSH (David T S Hayman), GCG (Guy-Crispin Gembu), IRP (Iñaki Rodriguez-Prieto), IVK (Ivan Kuzmin), KSB (Kate S Baker), TL (Tiziana Lembo), JJ (Javier Juste), DJL (DJ Long).
Samplingdate	Sampling date yyyy-mm-dd
Birthdate	Estimated date for start of the seasonal birth pulse for that location.
Mths.since.birthing	Months (to one decimal place) from the date of sampling to the beginning of the previous birth pulse
Cont_island	Whether sampling location is on the African continent or on an island
Country	Country of sampling location.To facilitate analysis, islands are recorded under their island name. The islands São Tomé and Príncipe are part of the country ‘São Tomé and Príncipe’ and Bioko, Rio Muni and Annobón are part of the country ‘Equatorial Guinea’
Region	Sampling region/city
Location	Specific sampling location
Latitude	Latitude in decimal notation
Longitude	Longitude in decimal notation
Bat.wt	Bat weight in grams, measured to the nearest gram
Sex	Bat sex. F (Female), M (Male), NA (not recorded)
Age	Age classification, assessed by morphological characteristics. N (Neonate; <2mths), J (Juvenile; 2 – <6 months), SI (Sexually Immature; 6 – <24 months) or A (Adult; ≥24 months).
Age.3	Age classification, assessed by morphological characteristics and timing of sampling relative to the birth pulse. For a subset of samples, the timing of sampling in relation to the birthing season permitted further classification of SI individuals into 6-month age groups SI.1, SI.2 and SI.3 (6 – <12, 12 – <18, 18 – <24 months, respectively).
Teeth.Age	Age in years from histological examination of tooth cementum annuli (0–15)
TeethCC	Teeth certainty score. Each age estimation was scored with a certainty code: A: highest certainty of reported age, B: histological evidence supported a given age result, or C: tooth or section quality was too compromised to accurately age.
Teeth.Age.Range	The possible age range based on teeth age and teeth certainty score.
Teeth.Age.mths	Teeth Age converted into months using time since estimated beginning of previous birth pulse (0–188)
Age.mths	Age in months, from ‘known age from teeth’ plus from individuals <2 years for which an age in months could be calculated from Age.3 classifications (0–188)
Repro.status	Reproductive status of adult female bats, and primiparous SI bats. NR (non-reproductive), P (pregnant), VEP (pregnant), EP (pregnant), MP (pregnant), LP (pregnant), L (lactating) or U (unknown) according to the descriptions provided in Table 2. Males and neonate, juvenile and other sexually immature females are recorded as NA.
Mother.ID	Sample ID of the mother if sample was a neonate or foetus (if known)
Offspring.ID	Sample ID of the neonate or foetus if sample was a pregnant or lactating bat (if known)
Forearm	Forearm length in millimetres, measured to one decimal place
Band.no.	ID number on metal thumb bands attached to some bats
LBV.mFAVN	Result from Lagos Bat Virus mFAVN serological assay. 0 (seronegative), 1 (seropositive), NA (no result)
Henipavirus	Result from henipavirus Luminex binding assay, using a cutoff so that samples above the cutoff are 99% likely to be seropositive. Data is from the Nipah virus assay, but is presumed to represent cross-reactivity to African henipaviruses rather than the presence of Nipah virus itself. 0 (seronegative), 1 (seropositive), NA (no result)
AchPV1	Result from Achimota virus 1 virus neutralisation assay. 0 (seronegative), 1 (seropositive), NA (no result)
AchPV2	Result from Achimota virus 1 virus neutralisation assay. 0 (seronegative), 1 (seropositive), NA (no result)
GeneticsID	Alternative sample ID used in genetic analyses
Cytb	Cytochrome *b* sequence
GenBankAccession	Accession number for cytochrome *b* sequences deposited in genbank (PopSets 426207275 and 723591518)
T.1/T.2	Microsatellite allele sizes at loci T
S.1/S.2	Microsatellite allele sizes at loci S
F.1/F.2	Microsatellite allele sizes at loci F
W.1/W.2	Microsatellite allele sizes at loci W
N.1/N.2	Microsatellite allele sizes at loci N
Q.1/Q.2	Microsatellite allele sizes at loci Q
X.1/X.2	Microsatellite allele sizes at loci X
P.1/P.2	Microsatellite allele sizes at loci P
K.1/K.2	Microsatellite allele sizes at loci K
Ac.1/Ac.2	Microsatellite allele sizes at loci Ac
Af.1/Af.2	Microsatellite allele sizes at loci Af
Ai.1/Ai.2	Microsatellite allele sizes at loci Ai
Ad.1/Ad.2	Microsatellite allele sizes at loci Ad
Y.1/Y.2	Microsatellite allele sizes at loci Y
Ag.1/Ag.2	Microsatellite allele sizes at loci Ag
Ah.1/Ah.2	Microsatellite allele sizes at loci Ah
B.1/B.2	Microsatellite allele sizes at loci B
M.1/M.2	Microsatellite allele sizes at loci M

**Table 5:** Measures adopted to minimise and allow assessment of errors which may occur during the sampling, DNA extraction, amplification, sequencing, genotyping and data analysis processes (adapted from Bonin *et al.* 2004; Table 4).

**MEASURES ADOPTED TO REDUCE AND DETECT ERRORS**
Sampling
•Standard protocols followed for collection and labelling samples
•Sampling information logged on paper sampling sheets, and retained as backup
•Sampling data transferred to a single sampling database
DNA extraction
•Inclusion of negative controls to monitor contamination
•Consistency in extraction protocol
•Extraction performed in a different room to downstream analyses
DNA amplification
•Inclusion of negative control to monitor contamination
•Inclusion of positive control as a reference sample
•Consistency in amplification protocol
•Replicate amplifications - by main user, and also another user (blinded)
Sequencing
•Inclusion of negative control to monitor contamination
•Inclusion of positive control as a reference sample
•Consistency in sequencing protocol
•Sequencing in both forward and reverse directions
•Replicate sequencing - including multiple samples individually repeated, and a single sample repeated many times
Sequencing Analyses
•Alignment of forward and reverse sequences, with automated and manual checking of inconsistencies
•Alignment against GenBank database
•Multiple alignment - manual checking of all polymorphisms
Genotyping
•Perform pilot study to assess suitability of new loci for larger study
•Automated scoring, with manual checking
•Inclusion of positive control as a reference sample
•Discard low-quality samples
•Inclusion of a locus invariant in 99% of samples to monitor inter-assay variability of CEQ allele migration
•Cross-reading of datasets, and repeat scoring of overall dataset if required
Genotyping Analyses
•Discard poor markers from pilot study
•Quantify overall genotyping error rate and assess acceptability
•Consider genotyping errors as a possible cause of Hardy-Weinberg or linkage disequilibrium

**Table 6:** Mitochondrial DNA PCR repeat rates and sequencing error rates.

	**cytb original primers**	**cytb new primers**
Number of samples	207	438
Number of samples repeat PCR and sequenced	28	1
Number of duplicates run per sample	1	70
Total number of repeats	28	70
% of sequences repeated	13.5%	16.0%
Number of base pairs in sequence	418	397
Number of repeated sequences	28	70
Number of base pairs compared	11704	27790
Number of sequence errors (mismatches)	1	0
% PCR/sequence error (No of mismatches/no bp compared)	0.01%	0%
% samples extracted in duplicate, cytb PCR and sequenced	2.4%	
Number of errors (mismatches)	0	
% error	0.0%	

## References

[d1] Dryad Digital Repository Peel A 2016 http://dx.doi.org/10.5061/dryad.2fp34

